# Gastric calcifying fibrous tumor suspected to be complicated with immunoglobulin G4-related disease treated by laparoscopy and endoscopy cooperative surgery: a case report

**DOI:** 10.1186/s40792-019-0714-6

**Published:** 2019-10-22

**Authors:** Ryoga Hamura, Tomoki Koyama, Masahiko Kawamura, Takeshi Kawamura, Mayo Nakamura, Katsuhiko Yanaga

**Affiliations:** 1 Department of Surgery, Kawamura Hospital, Shizuoka, Japan; 2 Department of Surgery, The Jikei University School of Medicine, 3-25-8, Nishi-Shinbashi, Minato-ku, Tokyo, 105-8461 Japan; 3 Department of Pathology, The Jikei University School of Medicine, Tokyo, Japan

**Keywords:** Calcifying fibrous tumor, IgG4-related disease, LECS

## Abstract

**Background:**

Calcifying fibrous tumor (CFT) is a rare benign soft tissue lesion.

**Case presentation:**

A 30-year-old woman was admitted to our hospital with complaints of epigastralgia. A 15-mm submucosal tumor was identified in the greater curvature of the superior body of the stomach by upper gastrointestinal endoscopy. Endoscopic ultrasonography revealed a hypoechoic lesion with an acoustic shadow consistent with calcification. Computed tomography showed a gastric tumor with calcification. A gastrointestinal stromal tumor was diagnosed, and gastric wedge resection was performed by laparoscopy and endoscopy cooperative surgery. On pathological examination, the tumor was identified to be a CFT. Postoperative serum IgG4 levels were 26.0 mg/dl, which supported the diagnosis of probable immunoglobulin G (IgG) 4-related disease, according to the comprehensive diagnostic criteria of IgG4-related disease. The patient was discharged on postoperative day 7 and remains well with no evidence of tumor recurrence for 2 years after resection.

**Conclusion:**

We herein reported a patient with a gastric CFT suspected to be complicated with immunoglobulin G4-related disease that was successfully treated by laparoscopy and endoscopy cooperative surgery.

## Background

Calcifying fibrous tumor (CFT) is a rare benign mesenchymal tumor which usually occurs in the limbs, trunk, and deep soft tissue [1, 2]. Gastrointestinal tract CFTs are very rare, and most cases are incidentally detected by endoscopy. Recently, gastrointestinal CFT has been thought to be a gastrointestinal lesion of immunoglobin 4 (IgG4)-related disease [3, 4]. For CFTs, surgical or endoscopic resection and postoperative follow-up for recurrence are recommended. We herein report a gastric CFT treated by laparoscopy and endoscopy cooperative surgery (LECS) with good outcomes.

## Case presentation

**Fig. 1:**
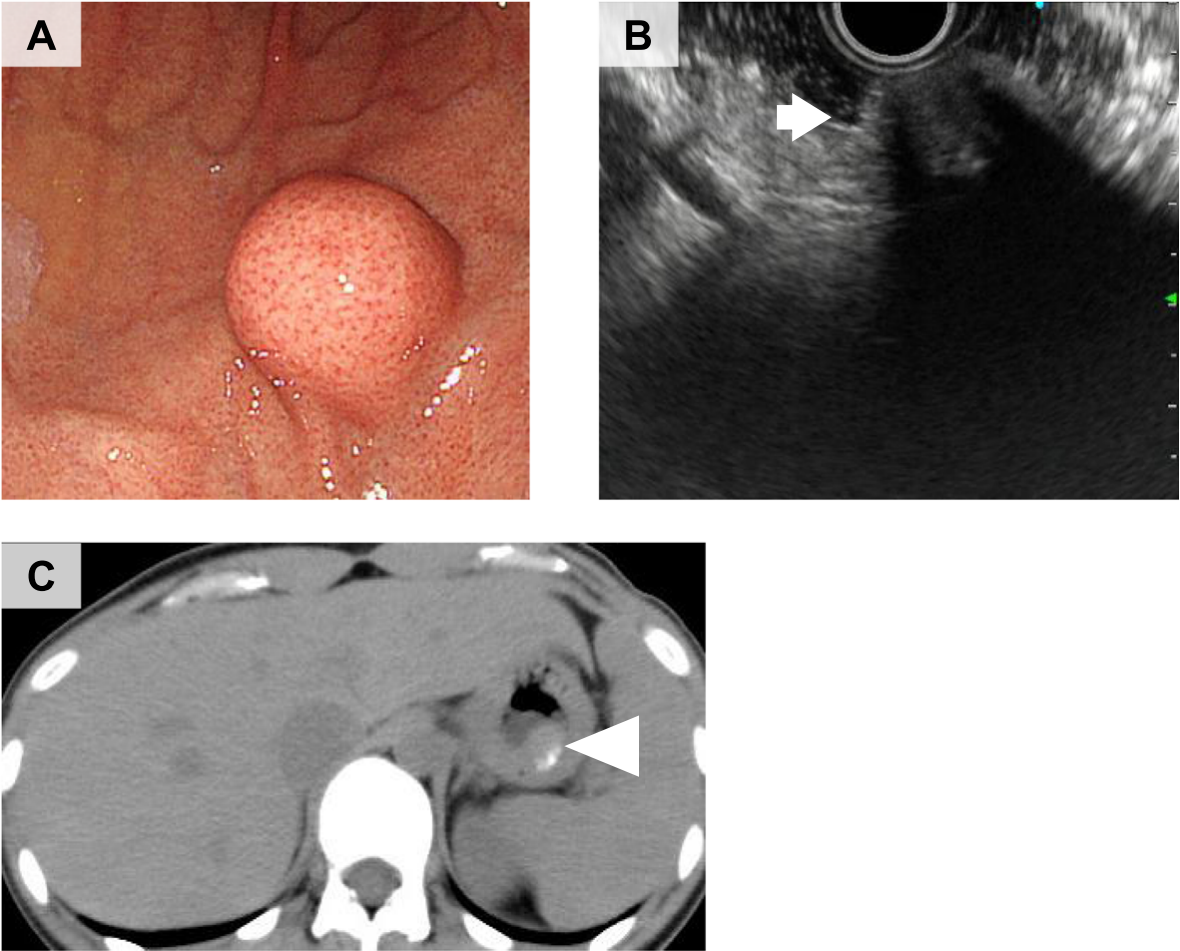
Upper gastric endoscopy showed a submucosal tumor in the gastric body (**a**). Endoscopic ultrasonography demonstrated a heterogeneous hypoechoic lesion with a shadow (**b**, arrow), consistent with calcification. Computed tomography showed calcifying gastric mucosal tumor (**c**, arrowhead)

**Fig. 2:**
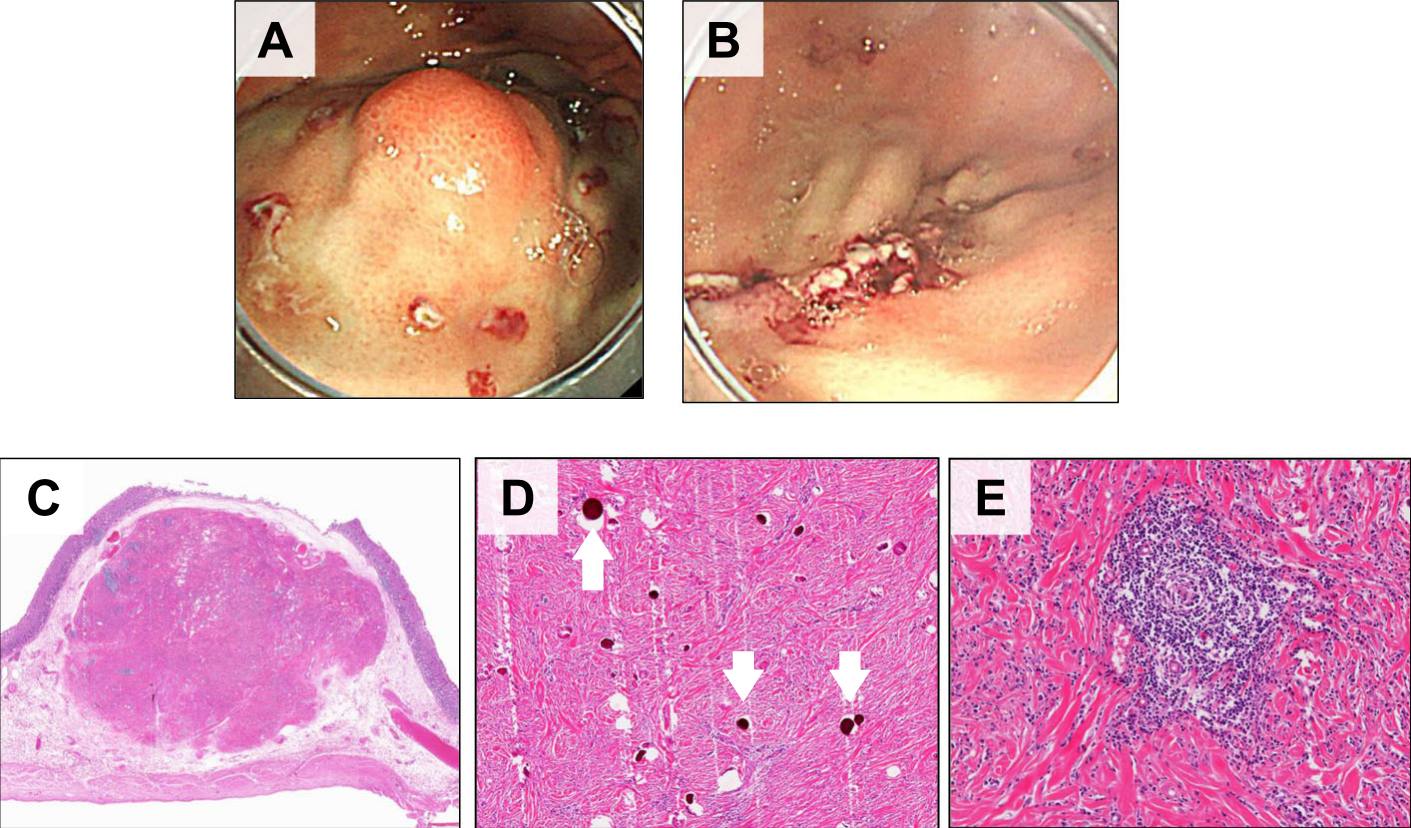
The endoscopic margin was 5 mm around the tumor (**a**), and we performed curative tumor resection (**b**). Macroscopically, the resected specimen showed a gastric submucosal tumor (20 × 18 × 16 mm) (**c**). Pathological examination of the tumor revealed psammoma bodies (**d**, arrows), spindle cell proliferation with abundant hyalinized collagen, and infiltration of lymphoplasmacytic cells in the tumor (**e**)

**Fig. 3:**
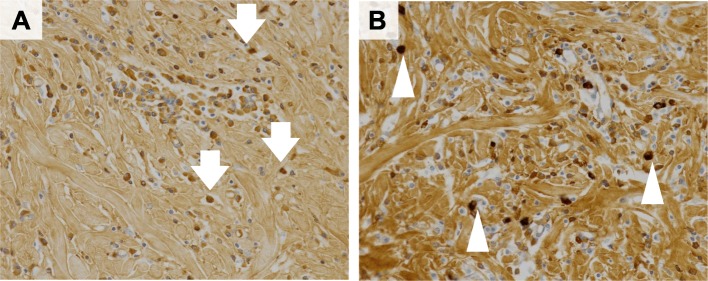
Immunological examination showed IgG-positive cells (**a**, arrows) and IgG4-positive cells (**b**, arrowheads) in the gastric tumor

A 30-year-old woman was admitted to our hospital with epigastralgia. Upper gastrointestinal endoscopy revealed a 15-mm submucosal tumor in the greater curvature of the superior body of the stomach (Fig. 1a). Endoscopic ultrasonography (EUS) showed a hypoechoic lesion with an acoustic shadow consistent with calcification (Fig. 1b). The laboratory data were within normal ranges. Computed tomography (CT) revealed a calcifying gastric submucosal tumor (Fig. 1c). Periodic upper gastrointestinal endoscopy was performed based on the absence of apparent malignancies such as ulcer or mucosal irregularity and small tumor size. One year later, the tumor appeared to have enlarged by upper gastrointestinal endoscopy, and gastrointestinal stromal tumor (GIST) was suspected. Based on Japanese clinical practice guidelines for GIST [5], surgical intervention was considered. Therefore, gastric wedge resection was performed via LECS. The endoscopic resection margin was 5 mm around the tumor (Fig. 2a, b). Operation time was 100 min and intraoperative blood loss was negligible. The resected specimens exhibited findings indicative of gastric submucosal tumor. Pathological examination demonstrated psammoma bodies, spindle cell proliferation with abundant hyalinized collagen, and infiltration of lymphoplasmacytic cells (Fig. 2c–e). Immunohistochemical studies were negative for CD117 (C-kit), α-smooth muscle actin, S100, desmin, and CD34-positive cells. Therefore, the tumor was diagnosed as CFT. Furthermore, IgG4-related disease was suspected because IgG-positive cells were involved with the IgG4 to IgG ratio of 54.6%, and IgG4-positive plasma cells were detected at 37 per HPF in the tumor (Fig. 3a, b). The postoperative serum IgG4 levels were 26.0 mg/dl, which supported the diagnosis of probable IgG4-related disease, according to the comprehensive diagnostic criteria for IgG4-related disease [6]. After surgery, the patient showed satisfactory recovery and was discharged on postoperative day 7. Two years later, the patient showed no evidence of recurrence, when examined by endoscopy.

## Conclusions

CFT was reported first as a rear benign soft tissue tumor in children by Rosenthal and Abdul-Karim [1, 2], which was previously named as “calcifying fibrous pseudotumors” as an abnormal reaction in the healing process of tissues [7]. The World Health Organization (WHO) established the name for this lesion in 2002 as “CFT” in the newly published classification of tumors of soft tissue and bone [8]. CFT has female predominance (*M*:*F* = 1:1.27) and is typically found between 20 and 30 years of age [2]. Gastrointestinal CFTs, including stomach and small intestine, are rare [9–13]. Most cases with gastrointestinal CFTs are asymptomatic. The cause of CFT is thought to be related to previous infection, history of trauma, and surgical intervention; however, the definitive mechanism or causes have not been confirmed. A relationship to genetic factors was suspected because of familial CFTs [14]. Although gastrointestinal CFTs are benign mesenchymal tumors for which local resection may be sufficient, they are difficult to distinguish from gastrointestinal submucosal tumors such as GIST, schwannomas, and leiomyomas [2, 15]. Furthermore, CFT does not metastasize and has a low risk of recurrence [2, 16, 17]. Histological characteristics of CFT include calcifying psammoma bodies in abundant dense fibro-collagenous tissue, collection of lymphocytes, and occurrence of plasma cells [2, 7, 15].

Recently, thickening of the gastrointestinal wall and an IgG4-related pseudotumor have been considered to suggest a gastrointestinal lesion of IgG4-related disease [3, 4]. IgG4-related disease is diagnosed in the presence of diffused/localized swelling or masses in a single or multiple organs with the elevation of serum IgG4 levels more than 135 mg/dl, or for histological findings of abundant infiltration of IgG4-positive plasma cells and lymphocytes along with fibrosis [6]. In the current case, although IgG4-positive cells were found on pathological examination, postoperative serum IgG4 levels were normal (26 mg/dl). Some reports show IgG4 positive cells in gastrointestinal CFTs, but IgG4-related disease was not confirmed [3]. Nevertheless, it is important to suspect IgG4-related disease and to monitor the serum IgG4 levels in such cases.

In our case, the preoperative differential diagnosis of SMT was a non-epithelial mesenchymal tumor such as GIST and leiomyoma based on endoscopic findings and EUS appearance. As a preoperative diagnosis, GIST with calcification was highly suspected. Then we performed gastric wedge resection via LECS. LECS is considered a good adaptation for small SMTs, although gastric wedge resection is a standard treatment for GIST [18]. Since CFT is a benign tumor, the risk of metastasis or recurrence is limited. However, the recurrence of cervical CFT has been reported [2, 16, 17]. For curative resection of CFT, sufficient surgical margins in surgery are necessary. When CFT is suspected, the patient should be tested for IgG4-related disease. The relation of IgG4-related disease and IgG4-related pseudotumor should be analyzed in large studies. The patient in this report remains well with no evidence of tumor recurrence for 2 years after resection. To the best of our knowledge, this is the first report that has described using LECS to treat a gastric CFT.

In conclusion, we herein reported a gastric CFT treated by laparoscopy and endoscopy cooperative surgery that was suspected to be a IgG4-related disease.

## Data Availability

Data sharing is not applicable to this article as no datasets were generated or analyzed during the current study.

## Abbreviations

CFT: Calcifying fibrous tumor
CT: Computed tomography
GIST: Gastrointestinal stromal tumor
IgG: Immunoglobulin G
LECS: Laparoscopy and endoscopy cooperative surgery
